# Mitochondrial Calcium Uniporter Structure and Function in Different Types of Muscle Tissues in Health and Disease

**DOI:** 10.3390/ijms20194823

**Published:** 2019-09-28

**Authors:** Nadezhda V. Tarasova, Polina A. Vishnyakova, Yulia A. Logashina, Andrey V. Elchaninov

**Affiliations:** 1 Institute of Molecular Medicine, Sechenov First Moscow State Medical University, Trubetskaya str. 8, bld. 2, Moscow 119991, Russia; yulia.logashina@gmail.com; 2 National Medical Research Center for Obstetrics, Gynecology and Perinatology Named after Academician V.I. Kulakov of Ministry of Healthcare of Russian Federation, 4 Oparina Street, Moscow 117997, Russia; vpa2002@mail.ru (P.A.V.); elchandrey@yandex.ru (A.V.E.); 3 Shemyakin-Ovchinnikov Institute of Bioorganic Chemistry, Russian Academy of Sciences, Miklukho-Maklaya Street 16/10, Moscow 117997, Russia; 4 Scientific Research Institute of Human Morphology, 3 Tsurupa Street, Moscow 117418, Russia; 5 Peoples’ Friendship University of Russia, 6 Miklukho-Maklaya Street, Moscow 117198, Russia

**Keywords:** calcium, calcium signaling, mitochondrial calcium uniporter, calcium homeostasis, regulatory pathways, myocardium, skeletal muscle, smooth muscle

## Abstract

Calcium ions (Ca^2+^) influx to mitochondrial matrix is crucial for the life of a cell. Mitochondrial calcium uniporter (mtCU) is a protein complex which consists of the pore-forming subunit (MCU) and several regulatory subunits. MtCU is the main contributor to inward Ca^2+^ currents through the inner mitochondrial membrane. Extensive investigations of mtCU involvement into normal and pathological molecular pathways started from the moment of discovery of its molecular components. A crucial role of mtCU in the control of these pathways is now recognized in both health and disease. In particular, impairments of mtCU function have been demonstrated for cardiovascular and skeletal muscle-associated pathologies. This review summarizes the current state of knowledge on mtCU structure, regulation, and function in different types of muscle tissues in health and disease.

## 1. Introduction

Calcium ions (Ca^2+^) are indispensable for signal transduction and regulation of target cell activity. Mitochondria are widely recognized as one of the key organelles maintaining Ca^2+^ homeostasis [1,2]. In mitochondrial matrix, Ca^2+^ stimulates mitochondrial activity, metabolism, and energy production, thus allowing the cells to adapt to immediate metabolic needs [3]. On the other hand, the mitochondrial Ca^2+^ buffer prevents the excessive increase in metabolism induced by Ca^2+^ stimulation [4]. In addition to its influence on bioenergetics, mitochondrial Ca^2+^ plays an important role in the regulation of various aspects of the cell life from contractility [5], chemotaxis, and migration [6,7] to reactive oxygen species (ROS) production [8], cell cycle and proliferation, the mitochondrial permeability transition pore (mPTP) opening, and cell death [9,10,11].

Since Ca^2+^ is a universal second messenger, the mitochondrial Ca^2+^ homeostasis is affected in various pathologies. Indeed, alterations in mitochondrial Ca^2+^ handling were observed in a range of cancers [4,12], as well as cardiovascular [13] and neurodegenerative [14] diseases.

Ca^2+^ penetrates through the outer mitochondrial membrane via a voltage-dependent anion channel (VDAC) [15]. The mitochondrial Ca^2+^ uniporter (mtCU), located in the inner mitochondrial membrane, unequivocally represents the dominant mechanism of Ca^2+^ transport from intermembrane space to mitochondrial matrix, although several mtCU-independent mechanisms were identified as well [16]. Ca^2+^ efflux from mitochondria occurs via Na^+^/Ca^2+^ and H^+^/Ca^2+^ exchangers [1]. 

In this review, we discuss the most recent discoveries on the molecular structure of mtCU and the regulatory mechanisms underlying its activity in connection with its function in different types of muscle tissue under normal physiological conditions and in pathology.

## 2. Structural and Electrophysiological Characteristics of MtCU 

**Figure 1:**
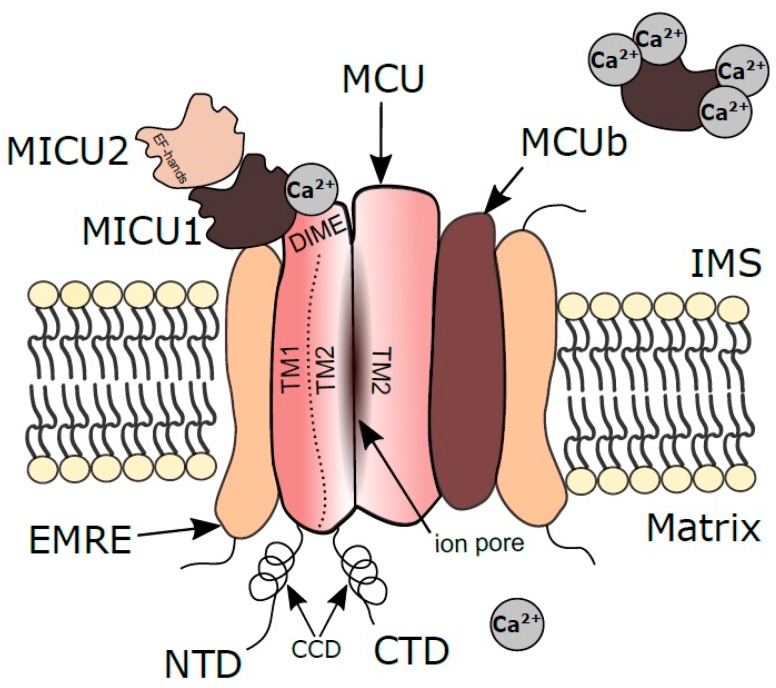
Scheme of mtCU structure. Mitochondrial calcium uniporter pore-forming subunit (MCU) and regulatory subunits are shown: MICU1 and 2—mitochondrial calcium uptake 1 and 2; MCUb—mitochondrial calcium uniporter dominant negative beta subunit; EMRE—essential MCU regulator. IMS—intermembrane space; NTD—N-terminal domain; CTD—C-terminal domain; TM1, TM2—two helices of the transmembrane domain; CCD—coiled coil domain; DIME—amino acids sequence mediating a Ca^2+^-modulated electrostatic interaction between MCU and MICU1. EF hand—helix-loop-helix structural motif of calcium-binding proteins MICU1 and MICU2. Modified from 10.1038/s41598-019-41996-3 under CC-BY.

MtCU consists of at least four main components: The pore-forming subunit (MCU) capable of higher-order oligomerization, the essential MCU regulator (EMRE), and two membrane gate-keeping factors (MICU1 and MICU2) (Figure 1) [17,18]. The summer of 2018 could rightly be called the season of cryo-electron microscopy (cryo-EM) for mtCU, since three significant works on its structure were published at this time [19,20,21]. Two years earlier, a core region of MCU was determined using nuclear magnetic resonance (NMR) by Oxenoid et al. [17]. MCU forms an elongated tetramer where each of the four protomers consists of three structural domains: Hydrophobic transmembrane domain (TMD), mainly located in the inner membrane; N-terminal domain (NTD) and coiled-coil domain (CCD), jointly forming a large hydrophilic region located in the mitochondrial matrix [19]. TMD is formed by two helices (TM1, TM2) which play a major role in the mtCU architecture: TM1 is essential for the interaction with EMRE while TM2 forms the central ion conduction pore [21]. A highly-conserved sequence motif W-D-Φ-Φ-E-P-V-T-Y (Φ stands for a hydrophobic amino acid) is located in the N-terminal region of TM2. The Asp and Glu residues in this motif from each of the four protomers form two acidic rings in the channel pore and has been proposed to constitute the selective Ca^2+^ filter [17,22,23,24]. Human MCU also contains the linker helix domain (LHD) that links the NTD and CC1 helix of CCD. In human MCU, the Asp and Glu residues in the W-D-I-M-E-P sequence motif (denoted as “DIME sequence”) are also involved in the formation of two rings of acidic residues for the selective Ca^2+^ permeation [18,25]. 

MtCU had been identified as a highly selective Ca^2+^ channel in the mitochondrial inner membrane long before its molecular nature was determined [12,26,27]. Nevertheless, the data on the electrophysiological properties of mtCU are rather heterogeneous and sometimes controversial. Whereas mtCU currents recorded in recombinant MCU-MICU1 [28] and MCU-EMRE [29] complexes in the planar lipid bilayers differ several times, data from whole mitoplasts differ by two orders [30,31]. To overcome the complexity of mitoplasts technique, a system based on *Xenopus* oocytes with the human MCU-EMRE complex targeted to the plasma membrane has been recently established [32]. Complex expression was confirmed by inhibitory analysis of inward Ca^2+^ current with mtCU selective inhibitor Ru360 [32]. Although this system can be considered as a promising tool for electrophysiological analysis both to determine fundamental molecular mechanisms and to test new potential pharmacological modulators, the reported mtCU-current parameters would require additional validation before the introduction of the model to applied research.

MtCU is better investigated as the assembled complex rather than individual subunits. This circumstance, however, does not interfere with effective simulation of the mtCU activity. Wacquier et al. introduced the computational model based on experimental data, which successfully described Ca^2+^ kinetics in either a suspension of mitochondria isolated from hepatocytes or the mitochondria in intact hepatocytes and, importantly, accounted for the difference between these two systems [33]. In addition, they analyzed the Ca^2+^ currents with consideration of the mitochondrial heterogeneity: The authors assumed that the total mitochondrial volume might correspond to different numbers of individual mitochondria. This consideration is important because the Ca^2+^ influx strongly correlates with the level of MCU expression and protein abundance in individual organelles. Despite the availability of the experimental data sufficient to create a computer model of Ca^2+^ currents via mtCU, many questions about the structure and mutual orientation of the subunits in the complex are still unclear. Next, we will focus on the structural and regulatory features of the human mtCU components.

### 2.1. MCU

The human MCU monomer consists of 351 amino acids and weighs about 40 kDa. Most of the data on the MCU tetramer structure is obtained by the cryo-EM approach and is particularly related to the structural features of the pore-forming tetramer channel discussed above. In this section, we will consider structural features and the role of extra-membrane parts of the tetramer in the functional activity of the whole complex. The matrix part of the tetramer protrudes 70 Å from the membrane [20], and has four “legs” extending from TMDs, formed by parts of NTD, CCD, and LHD (in the case of humans) [18]. The space between the legs allows the ions to diffuse into the matrix after passing through the pore [20]. The N-terminal matrix domain of human MCU consists of a conserved β-grasp-like fold containing a high-density cluster of negatively charged residues, the so-called MCU-regulating acidic patch (MRAP) [34]. Interaction of these residues with divalent cations (Ca^2+^ or Mg^2+^) destabilizes the tetramer toward monomer, thus causing inhibition of the mtCU activity in response to Mg^2+^ and Ca^2+^ binding [34].

Another structural feature of MCU is post-translational modifications which affect the functional activity of the protein. Cys is highly sensitive amino acid incorporated into proteins in the thiol (R-SH) form, which subsequently becomes oxidized or converted into disulfides (R-S-S-R). The post-translational modifications of Cys include disulfide formation, S-glutathionylation, S-nitrosylation, persulfidation, etc [35]. Its pronounced oxidizability makes Cys a redox-sensor in the cell. Dong et al. identified a conserved cysteine at position 97 in the MCU’s NTD as a reactive thiol susceptible to S-glutathionylation upon oxidative stress. The authors found that the MCU oxidation promotes the MCU higher-order oligomer formation while increasing the rates of Ca^2+^ uptake, elevating mitochondrial concentrations of ROS, and enhancing the Ca^2+^-induced cell death [35]. NTD is also involved in the interaction of MCU with Miro1, the outer mitochondrial membrane protein important for mitochondrial movement. Niescier et al. revealed specific interaction of Miro1 with the MCU’s NTD and considered it as a link in novel mechanism modulating the mitochondrial Ca^2+^ uptake and mitochondrial transport [36].

The opposite part of the MCU tetramer faces the intermembrane space and acts as an important site of interaction with the MICU1 regulatory subunit, although whether this interaction occurs inside the membrane or in the intermembrane space is still debatable. As recently shown by Phillips et al., Asp in the MCU’s DIME sequence mediates Ca^2+^-modulated electrostatic interaction with MICU1 [24]. In the absence of Ca^2+^, MICU1 binds to DIME with two arginines and blocks the intermembrane space entrance of the MCU pore. In the presence of Ca^2+^, its ions bind to MICU1 and disrupt this interaction, leading to the pore opening [24,37]. Importantly, MICU1 expression is related to MCU level, and the ratio of MICU1 and MCU protein levels positively correlates with their binding, which indicates the importance of the Ca^2+^ threshold for Ca^2+^ uptake and cooperativity of the whole mtCU complex [38].

### 2.2. MCUb

MCUb is a protein of 336 amino acids and has a molecular weight of about 40 kDa (similar to MCU). The sequence identity between MCUb and MCU is 48.77% (according to BLAST). MCUb alone is incapable of forming a Ca^2+^-conductive channel but plays an important role in the regulation of mtCU functional activity by forming heteromers with MCU [39,40]. An increase in the MCUb-to-MCU ratio has been shown to reduce the activity of MCU; thus, MCUb acts as a dominant-negative subunit that attenuates the Ca^2+^ currents via the channel [39].

### 2.3. MICU1

The regulatory subunit MICU1 consists of 476 amino acids and weighs about 54 kDa. As we have already mentioned, MICU1 binds with MCU through the DIME sequence thus serving as a gatekeeper of mtCU [37,41]. At low Ca^2+^ concentrations, MICU1 inhibits the Ca^2+^ current; at high Ca^2+^ concentrations, the MCU-binding site in MICU1 is blocked by Ca^2+^ ions and the pore conductance is restored. MICU1 is able to form a homodimer or heterodimer (by disulfide bonds) with another regulatory subunit—MICU2. Both MICU1 and MICU2 have high affinities for Ca^2+^ [42]. When a heterodimer is formed, the Ca^2+^ binding capacity of individual subunits (in the nanomolar range of concentrations) is preserved. As demonstrated by Kamer et al., the MICU1-MICU2 heterodimer selectively binds with liposomes containing cardiolipin; this finding suggests a mechanism for association of the complex with the inner mitochondrial membrane [42].

MICU1 has two structural domains separated by a long alpha helix [42]. Each domain contains a helix-loop-helix structural motif, the so-called EF-hand, typical for Ca^2+^-binding proteins. This motif is especially important for overall ion selectivity of the uniporter. Kamer et al. have suggested that specific metal-ligand interactions in EF-hand underlie the ability of MICU1 to differentiate between Ca^2+^ and Mn^2+^ [43]. According to their model, at high Ca^2+^ levels, Ca^2+^ binds to EF hands promoting the channel opening and facilitating the passage of Ca^2+^ and Mn^2+^ through the pore. When manganese level is high and Ca^2+^ is low, Mn^2+^ binds to the MICU1`s EF-hands without structural changes in the MICU1-MCU interconnection and the pore remains closed. In MICU1 knockout cells (HEK-293T), MCU has no such ion selectivity, which leads to Mn^2+^ loading in mitochondria. Wettmarshausen et al. came to a similar conclusion: Co-expression of MCU and MICU1 in the yeast *S. cerevisiae* as a model system protects the cells against the uniporter-dependent Mn^2+^ overload and Mn^2+^ toxicity [44]. 

In vivo deletion of MICU1 leads to and larval lethality in *Drosophila* [45], while in mice causes Ca^2+^ overload, severe neurological and myopathic defects, similar to that observed in MICU1-deficient patients [41]. 

MICU1 degradation is Parkin-mediated and occurs in proteasome. Interestingly, it proceeds independently of the E3-ubiquitin ligase activity of Parkin protein but depends on its Ubl-domain [46]. Parkin is therefore considered as a regulator of mtCU composition and activity.

MICU1.1 is a splicing isoform of MICU1 having an extra micro-exon between exons 5 and 6, which encodes four amino acids and is highly conserved among all vertebrates [47]. MICU1.1 is expressed in skeletal muscle and at a lower level in the brain, and nowhere else, whereas the MICU1 expression is ubiquitous. All in all, MICU1.1 acts as an alternative regulator of mtCU in certain tissues; its function is believed to be related to the fast muscle contraction. 

### 2.4. MICU2

MICU2, another gatekeeper, contains 434 amino acids and has a molecular weight of about 50 kDa. Kamer et al. [48] recently revealed the presence of two structural lobes in murine MICU2 protein, with a pair of EF-hands each, described essential structural differences in N- and C-terminal segments between MICU2 and MICU1, and also proposed computational models for participation of the MICU1–MICU2 complex in gating mechanisms of the uniporter. Human MICU2 dimerizes in Ca^2+^ concentration-dependent manner in two types of dimers: Back-to-back or face-to-face dimers. At low Ca^2+^ levels, MICU2 forms back-to-back homodimers thus blocking the Ca^2+^ conduction through the pore, while at high Ca^2+^ levels both MICU2 and MICU1 form face-to-face dimers which do not interfere with Ca^2+^ currents [49]. Payne et al. showed that at low Ca^2+^ concentrations MICU1 mainly performs the gatekeeping function, while MICU2 modulates its inhibitory effect on mtCU [50]. Despite the seemingly subordinate role of MICU2 in the regulation of Ca^2+^ currents, depletion of *MICU2* decreases the levels of both MCU and MICU1 proteins without altering transcription of the corresponding genes [51]. MICU2 null mutations in patients lead to neurodevelopmental complications, including gliosis, periventricular haemorrhage, sagittal sinus thrombosis, periventricular encephalomalacia, and cognitive impairment [52]. Disruptions in Ca^2+^ homeostasis persisting in the patients’ cells: The MICU2-deficient cells exhibited mitochondrial Ca^2+^ overload at resting state and showed a slower bradykinin-stimulated Ca^2+^ influx than control cells from healthy individuals [52]. 

### 2.5. MICU3

MICU3 contains 530 amino acids and weighs about 60 kDa. According to UNIProt database, the MICU3 structure also has the EF-hand motif (as in MICU1 and MICU2), and its presence in skeletal muscle has been reported [53]. In earlier studies, MICU3 was considered as a regulatory subunit with gatekeeping function (similarly with MICU1 and MICU2) [49]. However, MICU3 has been recently shown to form heterodimers with MICU1 but not with MICU2 or itself, with prevalent expression of MICU3 in brain tissue [54]. Stimulation of MICU3-overexpressing HeLa cells with agonists (e.g., ATP) led to increased mitochondrial Ca^2+^ uptake rates. All in all, MICU3 is presently considered as mtCU activator with reduced gatekeeping function [54]. The presence of additional brain-specific mtCU regulators may be related to fine-tuning of Ca^2+^ signals in neuronal networks. 

### 2.6. EMRE

EMRE, also known as SMDT1, is a 107 amino acids protein weighing about 11 kDa. It contains a TMD while its N- and C- terminal domains are exposed to the matrix and intermembrane space, respectively [18,55,56]. A set of acidic residues exposed to mitochondrial matrix works as a Ca^2+^ sensor [57]. EMRE is only found in metazoans, and cryo-EM structural analysis of human MCU-EMRE complex demonstrated that each EMRE molecule binds to one MCU protomer, with the resulting architecture of the MCU-EMRE tetramer working as a minimal functional mtCU complex [18]. Without MCU, EMRE is ineffective and insufficient for Ca^2+^ uptake [55,58]. It is now clearly evident that EMRE provides both physical interconnection of MICU1 and MICU2 with MCU and stabilizes the entire Ca^2+^ pore in open state with β-hairpin in its N-domain [18,56]. EMRE participates not only in the regulation of single uniporters, but also in the functional coupling and coordination between several mtCU complexes [18]. Thus, EMRE is considered as a stabilizer of the MCU interaction with other regulatory subunits and a major regulator of mtCU cooperativity.

### 2.7. MCUR1 

MCUR1 contains 359 amino acids and weighs about 40 kDa. MCUR1 is a regulatory protein which is often overlooked in the context of mtCU functioning. Orthologs of MCUR1 are found in various organisms, but whether to accept MCUR1 as an independent and separate subunit is still questionable. The published data indicates that MCUR1 deletion significantly impairs Ca^2+^ uptake; therefore, MCUR1 may be considered as a positive regulator of mtCU complex [59]. Structurally, human MCUR1 has several domains: A head, a β-layer neck and a stalk, set side by side in the matrix, and a transmembrane domain [60]. The head domain of MCUR1 directly contacts the NTD of MCU, and MCUR1 function is controlled proteolytically [59,60,61]. Tissue-specific MCUR1 knockout (KO) in cardiac muscle- and endothelium lead to decreased ATP levels, impaired mitochondrial Ca^2+^ import, and increased autophagy in cardiomyocytes and endothelial cells [59]. MCUR1 also plays the essential role in the forming EMRE-MCU complex: In addition to its interaction with MCU, MCUR1 could directly interact with EMRE thereby providing a link to the full assembly complex. Up-regulated expression of MCUR1 was observed in a pathological condition: MCUR1 promoted the epithelial-mesenchymal transition of hepatocellular carcinoma cells followed by invasion and metastasis [62].

## 3. MtCU Regulation by Intracellular Signaling Pathways

The proper mitochondrial Ca^2+^ handling, provided by proper mtCU functioning, is essential for the well-being of individual cells and the whole organism. Fine-tuning of mtCU function is carried out at multiple levels, including transcriptional, post-transcriptional, and post-translational levels [63]. Several transcriptional regulators of mtCU are known, and most likely there are more of them to be described. The cyclic AMP response element-binding protein (CREB) was the first identified transcriptional factor regulating MCU expression [64]. Shanmughapriya et al. discovered a direct interaction of activated CREB with an MCU promoter stimulating the gene expression. The important point is that cytoplasmic Ca^2+^ signals induce CREB activation through its phosphorylation by various kinases. Damping of Ca^2+^ oscillations reduces CREB phosphorylation leading to significantly lower MCU expression [64]. Recently, the same group revealed downregulation of MICU1 expression by transcription factor Foxd1 in human induced pluripotent stem cells (hiPSCs) under glycolytic and hypoxic conditions. Foxd1 knockdown and the consequent MICU1 upregulation turned out to be necessary for the periodic cytosolic Ca^2+^ oscillations essential for cell differentiation and maturation [65].

Post-transcriptional regulation of mtCU expression and function by microRNAs (miRs) is not yet fully understood, although the influence of miRs on mitochondrial Ca^2+^ was recently reviewed [66]. Remarkably, all miRs, identified as mtCU regulators, reduce the MCU subunit expression, but the overall physiological effect depends on tissue type and other conditions. Indeed, miR-340 negatively regulates MCU expression and suppresses breast cancer metastasis [67]. At the same time, miR-25 overexpression in colon cancer ultimately resulted in decreased MCU protein expression providing protective anti-apoptotic effects and promoting cancer cells survival [68]. In the pulmonary arterial hypertension, miRs 25 and 138 cooperatively cause an impairment of mtCU function [69]. More specifically, simultaneous upregulation of miR-25 and miR-138 not only downregulates MCU transcription, but also inhibits MCU expression by means of binding with 3′-untranslated region of MCU transcripts. The reciprocally increased MICU1 expression further enhances the mtCU dysfunction. On the other hand, downregulation of these miRs or restoration of mtCU function accordingly restores the Ca^2+^ homeostasis [69]. It should be noted that some authors consider pulmonary arterial hypertension as a cancer-like disease [70], and a shared miR-dependent mechanism promoting both cancer and pulmonary arterial hypertension progression would support this theory. Surprisingly, the oxidative stimulation-induced miR-25 elevation inhibits MCU expression in cardiomyocytes, thus being cytoprotective [71]. The MCU protein expression in myocardium is also selectively repressed by miR-1 [72], the most abundant miR in the heart, belonging to the “myomiR” family with muscle-specific expression and recognized for the central regulatory role in muscle biology [73]. MiR-1 targets the MCU 3′-untranslated region and causes a marked reduction in the MCU protein content, while the MCU mRNA level remains unchanged. Consistently, miR-1 overexpression significantly reduced the amplitude of the steady-state mitochondrial Ca^2+^ level in cardiomyocytes during pacing and suppressed the hypertrophic gene expression program. The beta-adrenergic receptor/Akt/FOXO axis was proposed as an upstream regulator of miR-1/MCU pathway [72]. DRP1 protein, responsible for mitochondrial fission, was also proposed as an upstream regulator of miR1-mtCU cascade by some authors [74], indicating interconnection of some pathways at the point of mtCU. 

At the post-translational level of regulation, mtCU undergoes a variety of modifications including phosphorylation, S-glutathionylation, and methylation. It is now accepted that bioenergetic, oxidative, and Ca^2+^ pathways meet and regulate each other in mitochondria [8]. For this reason, the proteins involved in calcium handling and signaling require redox-sensors. In cell models of inflammation and hypoxia, the concomitant oxidative stress-induced S-glutathionylation of MCU leads to more prominent MCU oligomerization, an increase in mitochondrial Ca^2+^ uptake and ROS production, and, finally, cell death induced by the Ca^2+^ overload [35].

Phosphorylation of MCU by proline-rich tyrosine kinase 2 (Pyk2) after adrenoceptor stimulation in intact cardiac H9c2 myoblasts has been reported [75]. Adrenoceptor stimulation causes translocation of Pyk2 to mitochondrial matrix where Pyk2 directly phosphorylates MCU tyrosine residue(s), promoting MCU oligomerization into functional tetrameric MCU channels. The enhanced mitochondrial Ca^2+^ uptake via mtCU results in increased generation of ROS as well as calcium overload and initiation of the apoptotic signaling [75]. Similar activation of the Pyk2/MCU pathway accompanied by mitochondria damage and an increase in apoptosis was recently detected in the ischemic rat brain after middle cerebral artery occlusion, indicating that the Pyk2/MCU pathway may be a universal mediator of stress-induced mitochondrial damage and cell death [76].

Although direct phosphorylation of MCU by CaMKII in cardiac muscle was demonstrated, its relevance is still debatable. In 2012, Joiner et al. [30] published impressive data indicating the anti-ischemic effect of CaMKII inhibition with a transgenic mitochondrial-targeted inhibitory protein. The inhibition attenuated mitochondrial disruption, caused by ischemia and reperfusion, and protected cardiomyocytes from apoptosis, providing a significant decrease in the ischemic lesion area in myocardium. Physical association of CaMKII and mtCU was proved by their co-immunoprecipitation from the myocardial mitochondrial lysate, and the candidate phosphorylation sites in MCU were identified [30]. However, the electrophysiological data indicating that CaMKII promotes the mPTP opening and myocardial tissue death by increasing the mtCU current were not confirmed in more recent studies by other groups [31,77]. Furthermore, no functional change in the mtCU current after CaMKII application was observed, which suggests the lack of mtCU regulation by CaMKII [31]. The latest work of Nickel et al. provides comprehensive assessment of CaMKII role in the control of mitochondrial Ca^2+^ uptake, respiration, and ROS production during β-adrenergic stimulation and pacing in cardiomyocytes and isolated cardiac mitochondria from the CaMKII knockout mice. The authors pointed out that the results were inconsistent with any relevant role of CaMKII in the control of mitochondrial Ca^2+^ uptake [77]. It is also noteworthy that Nickel et al. failed to reproduce the experiments of Joiner et al. on isolated hearts as well as the CaMKII/mtCU co-immunoprecipitation. Therefore, the role of CaMKII in the recovery after ischemia-reperfusion injury in vivo and the direct interaction of CaMKII with mtCU in cardiac muscle are still questionable. Interestingly, the mtCU phosphorylation by CaMKII at the highly conserved Ser92 [30,78] stimulates mitochondrial Ca^2+^ uptake which further activates the CaMKII-mediated mtCU phosphorylation thus forming a positive feedback circuit, and triggers migration of vascular smooth muscle cells (VSMCs) [79]. This observation brings up the matter of tissue-specific mtCU regulation and may encourage further studies in this direction.

Mitosis-specific phosphorylation of MCU by AMPK, necessary for the restoration of ATP levels during cell division, was recently reported [80]. The AMPK-dependent MCU phosphorylation at Ser57 activates the mitochondrial Ca^2+^ entry leading to the intensification of mitochondrial respiration and energy production necessary for the proper spindle dynamics [80]. The receptor-interacting protein kinase 1 (RIPK1), taking part in cell survival/apoptosis pathways, was recently shown to bind MCU for the mitochondrial Ca^2+^ uptake induction and thus contribute to the development of colorectal cancer [81]. Zeng et al. elucidated a RIPK1-MCU physical interaction in colorectal cancer samples from patients and HT29 cells using co-immunoprecipitation and confocal microscopy. The authors also identified Lys377, a known ubiquitination site, as crucial for the RIPK1 interaction with MCU. Unfortunately, this study provides no information regarding the specific phosphorylation site in MCU [81]. 

The large body of data on the mtCU regulation, obtained from cancer cells and models, are highly relevant for the search of new therapies along with the general understanding of mtCU regulation and functioning. For instance, a recently described Akt kinase-mediated phosphorylation of MICU1 at the N-terminal region in cancer is the first demonstration of a phosphorylation event for the MICU1 subunit [82]. More specifically, the mitochondrial pool of active Akt is responsible for the MICU1 phosphorylation at Ser124, which affects the MICU1 processing and results in the MICU1-MICU2 dimer instability. The loss of the MICU1 inhibitory influence on mtCU leads to a significant increase in the basal Ca^2+^ level in the mitochondrial matrix, culminating in the ROS production and tumor progression [82]. A more comprehensive functional evaluation of this MICU1 modification under normal and pathological conditions would be relevant.

In addition to phosphorylation, the MICU1 gatekeeper function is also modulated post-translationally through its methylation by arginine methyl transferase 1 (PRMT1) at Arg455 [83]. Interestingly, although the methylation of MICU1 desensitized it for Ca^2+^ and reduced the mitochondrial Ca^2+^ uptake, UCP2 was shown to bind exclusively to the methylated form and to restore the mitochondrial Ca^2+^ uptake in the cells with pronounced PRMT1 activity [83]. Although the early data on the UCP2/3 engagement in the regulation of Ca^2+^ uptake through the inner mitochondrial membrane were contradictory [84,85], more recent studies have confirmed the regulatory effect of UCPs on mtCU [86,87]. 

Proper mitochondrial functioning requires the tightly controlled turnover of mitochondrial proteins. In particular, the mtCU assembly dynamics is maintained by m-AAA metalloproteases (ATPases associated with diverse cellular activities) [88]. Mitochondrial m-AAA proteases AFG3L2 and SPG7 rapidly digest the unassembled EMRE; however, after the EMRE incorporation into mtCU, its turnover is substantially inhibited. The excessive EMRE expression leads to the paradoxical Ca^2+^ leakage into mitochondria through the constitutively active MCU-EMRE subcomplex and the increased rates of Ca^2+^ uptake [89]. On the other hand, AFG3L2 loss leads to the facilitated mitochondrial Ca^2+^ overload followed by mPTP opening and neuronal death in murine model [90]. Interestingly, the functional loss of SPG7 led to similar changes, while physical loss was characterized by lower basal levels of mitochondrial Ca^2+^ and the increased Ca^2+^ retention capacity [91]. Considerably increased EMRE protein content and other major alterations in the mtCU stoichiometry and assembly observed in SPG7 KO cells [91]. At the body level, the SPG7 null mutation in *Drosophila* model caused shortened lifespan, progressive locomotor defects, increased sensitivity to chemical and environmental stress, and muscular and neuronal degeneration [92].

Summing up, these data indicate that mitochondrial Ca^2+^ uptake through mtCU is precisely regulated at multiple levels. Several health disorders associated with disturbances in mtCU functioning and involving different types of muscle tissues are discussed in upcoming sections. 

## 4. The Role of MtCU in Cardiac Muscle

### 4.1. MtCU in Normal Myocardium Function

Main function of the heart is to ensure blood supply to all organs and tissues. To provide it, myocardium undergoes constant rhythmic cycles of excitation followed by contraction (excitation–contraction coupling) based on the permanent Ca^2+^ release/uptake cycles in the cytoplasm and buffering organelles of cardiomyocytes [93]. Interestingly, in cardiomyocytes, the removal of Ca^2+^ from the cytoplasm after myocardial contraction is mostly provided by activities of the Na+/Ca^2+^ exchanger (NCX) in sarcolemma and the sarcoplasmic/endoplasmic reticulum Ca^2+^ ATPase (SERCA), whereas in non-muscle cells the Ca^2+^ buffering and termination of cytosolic Ca^2+^ signals mostly depend on the mitochondrial Ca^2+^ uptake [1]. Although the mitochondrial Ca^2+^ uptake in cardiomyocytes constitutes less than 1% and the mtCU current is surprisingly low as compared to other tissues [93,94], it is definitely a key player coordinating the balance between energy supply and demand (excitation–metabolism coupling) [3]. Precise mechanisms regulating the mitochondrial Ca^2+^ handling in cardiomyocytes were described during the last decade [13].

After determination of the mtCU molecular identity, its role in the cardiac muscle has been widely investigated by using genetic manipulations. The whole-body MCU KO mice displayed unaffected cardiac function both under basal and stress conditions [58]. On the other hand, mice with a short-term cardiac-specific MCU KO displayed unchanged phenotype under basal conditions but showed an impaired adaptive response to acute stress [95,96]. 

Ca^2+^ entry through the L-type Ca^2+^-channels in cardiomyocytes induces Ca^2+^ delivery from the sarcoplasmic reticulum (SR) ryanodine receptors (RyR2) leading to formation of Ca^2+^ microdomains nearby the SR-mitochondria junction and further Ca^2+^ signal transduction into mitochondria via mtCU [97]. It should be noted, however, that exact composition of Ca^2+^ microdomains in cardiomyocytes is not yet fully understood. Indeed, mtCU was initially reported to be homogeneously distributed in the mitochondrial inner membrane, as observed in intact ventricular cardiomyocytes of adult rabbit [98]. A more recent study on submitochondrial membrane fractions obtained from mouse and rat heart mitochondria revealed that mtCU are concentrated at the SR-mitochondria contact sites at the mitochondrial periphery to promote effective Ca^2+^ transport [99]. Along the same line, this scientific group later reported spatial segregation of the mitochondrial Ca^2+^ efflux via NCLX from the mitochondrial Ca^2+^ uptake via mtCU [100]. The lack of Ca^2+^ extrusion at the most Ca^2+^-exposed area serves for optimization of the Ca^2+^ signaling efficiency and minimization of the energy costs [100]. The controversy concerning the distribution of mtCU in the inner mitochondrial membrane may be explained by the difference in experimental approaches as well as by species- or age-specific characteristics of cardiomyocytes. Overall, it is still difficult to draw any definite conclusions, and further investigations are required.

The kinetics of mitochondrial Ca^2+^ concentration in adult ventricular cardiomyocytes during excitation–contraction coupling is also debatable [1]. Cao et al. discussed two models proposed for the Ca^2+^ dynamics in cardiac mitochondria [13]. According to the first model, Ca^2+^ concentration in cardiac mitochondria oscillates in a beat-to-beat manner, whereas the second model assumes gradual mitochondrial Ca^2+^ uptake. The authors emphasize that the mitochondrial Ca^2+^ dynamics, which may depend on the animal species and pacing frequency, needs further investigation [13]. 

### 4.2. MtCU in Cardiac Hypertrophy

**Figure 2:**
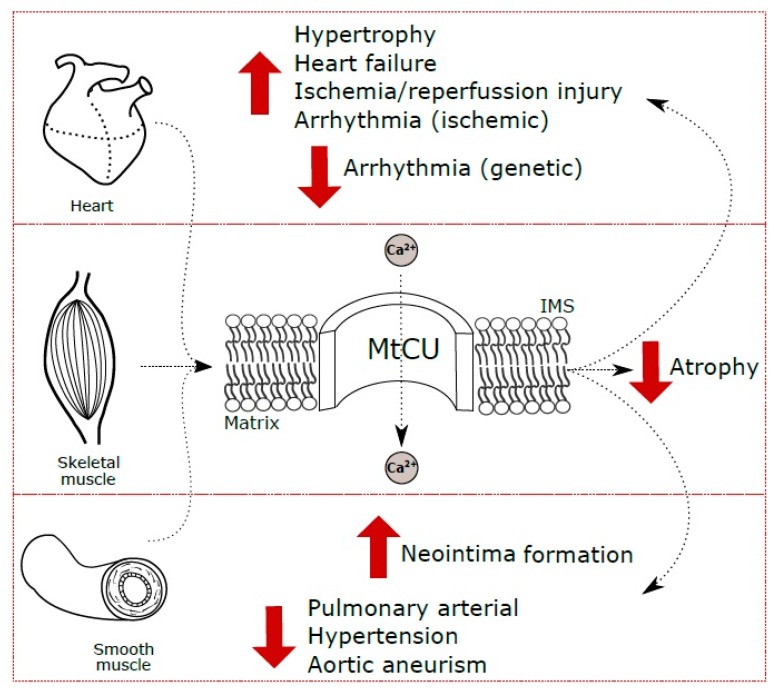
A summarizing scheme on the role of mtCU in pathological conditions of cardiac, skeletal and smooth muscle. Red arrows indicate the hyperfunction (up) or hypofunction (down) of mtCU complex associated with the pathology. IMS—intermembrane space.

Myocardium hypertrophy can be defined as an increase in the myocardial mass. It is well known, that in mammals the majority of cardiomyocytes lose the ability to proliferate shortly after the birth, and the subsequent heart growth proceeds mostly by an increase in the cardiomyocyte cell size. This is true under both normal physiological (growth, pregnancy, chronic exercise training) and pathological (hypertension, sarcomeric gene mutations, etc.) conditions [101]. Physiological cardiac hypertrophy during postnatal development is accompanied by both an increase in cell size and pronounced remodeling, including redistribution of mitochondria to the Ca^2+^ microdomains and SR maturation [102]. However, the data on the role of mtCU in cardiomyocyte maturation are extremely limited. Zaglia et al., for the first time demonstrated decreased MCU protein levels and the reciprocal miR-1 upregulation in the adult hearts as compared with the neonatal hearts in mice and humans, and concluded that the miR-1/MCU axis takes part in the postnatal cardiomyocyte maturation [72]. Increased cardiac pressure load during exercises or aortic stenosis caused a decrease in miR-1 and, accordingly, increased the MCU content indicating similar initial cardiomyocyte adaptation (Figure 2). However, the signs of local tissue damage and the substantially increased mRNA expression of the pore-inhibiting subunit MCUb were observed only under the pathological overload [72]. The increased MCU content in the ventricular myocardium during the pressure overload-induced hypertrophy is accompanied by autophagy inhibition and histological changes [103]. Therefore, although the pathological cardiac hypertrophy is compensated at the initial step, its further progression leads to decompensation and contributes to the development of severe cardiovascular disorders, such as arrhythmias and heart failure.

As expected, the mtCU inhibition under the overload-induced hypertrophy improved the cardiac function and mitigated the pathological changes [103]. On the other hand, the absence of MCU protein expression did not prevent the development of the overload-induced cardiac hypertrophy in MCU−/− mice [58]. This contradiction once again emphasizes the multifactorial etiology of cardiovascular diseases, as well as the necessity for precise regulation of mitochondrial Ca^2+^ homeostasis, reliably compensated in the case of congenital defects in corresponding genes.

### 4.3. MtCU in Arrhythmogenesis

Arrhythmias represent one of the primary causes of sudden cardiac death and a severe complication after acute ischemia and myocardial infarction [104,105]. 

Several heart diseases, including nonischemic heart failure and myocardial ischemia, are associated with increased arrhythmia risks and triggered activity. The ischemia-induced abnormalities in the ventricular automaticity are supposed to cause arrhythmias and attributed to the disturbed intracellular and, in particular, mitochondrial Ca^2+^ handling [106]. Mitochondrial Ca^2+^ uptake via mtCU modulates the spontaneous electrical activity of ventricular-like cardiomyocytes derived from mouse embryonic stem cells, together with RyR2 and IP3Rs at sarcoplasmic reticulum, while the plasmalemmal Ca-L-channels and NCX ensure the cell automaticity [107]. The beating rate of ventricular-like cardiomyocytes [107], as well as ventricular fibrillation after adrenergic stimulation in isolated hearts with pressure overload-induced hypertrophy [108], were substantially reduced after mtCU inhibition with selective inhibitor Ru360. On the other hand, direct stimulation of mtCU with natural plant flavonoid kaempferol exacerbated the mitochondrial Ca^2+^ accumulation and spontaneous premature ventricular contractions [108]. Consistent data were obtained on MCU knockdown mice and the mouse ventricular cell computer model and simulation [109]. In contrast, anti-arrhythmogenic effect was observed after mtCU activation in catecholaminergic polymorphic ventricular tachycardia (CPVT), a disease associated with mutation in RYR2 and characterized by episodes of life-threatening ventricular tachycardia upon the catecholaminergic stimulation after physical exercise or emotional stress [110]. Indeed, stimulation of mtCU with kaempferol led to enhanced mitochondrial Ca^2+^-uptake after the release of Ca^2+^ from SR and eliminated the arrhythmogenic Ca^2+^ waves induced by catecholaminergic stimulation in the CPVT murine cardiomyocytes [110]. These contradictions may originate from differences in both the experimental approaches and model objects. In particular, the embryonic stem cells-derived ventricular-like cardiomyocytes possess their own spontaneous electrical activity, while the adult ventricular cardiomyocytes do not. At the same time, arrhythmia during the pressure overload-induced hypertrophy is associated with excessive mechanical work of myocardium, while in CPVT it is caused by a genetic defect. 

Therefore, although the regulation of mitochondrial Ca^2+^ handling by mtCU is likely to provide a versatile mechanism for adjusting the electrical activity of pacemaker cells, the attempts to modulate it with pharmacological agents may lead to adverse consequences and should be studied in more detail. 

Disturbances in functioning of the mtCU regulators are also associated with the development of arrhythmias. For instance, *Ucp2−/−* mice have more pronounced susceptibility to the Ca^2+^ overload-induced arrhythmias and increased PRMT1 protein levels, which may be responsible for the decreased mitochondrial Ca^2+^-uptake in the *Ucp2−/−* hearts [111]. It is important to note that the UCP polymorphisms in humans are known to be associated with the heart rate variability [112]. Although the protective role of other mtCU regulators, Pyk2 and Akt, to some extent counteracts arrhythmogenesis [113,114], the mechanism has not yet been studied in detail and consideration of Pyk2 and Akt as anti-arrhythmogenic targets may require further investigation. 

### 4.4. MtCU in Myocardial Ischemia/Reperfusion Injury

Ischemic heart disease remains a major factor of death and disability worldwide [115], and the mitochondrial Ca^2+^ mishandling in cardiomyocytes during ischemia/reperfusion injury is well documented [116]. The cardiomyocyte death following ischemic episode is mainly caused by Ca^2+^ overload-induced ROS generation and mPTP opening.

The role of mtCU in the cardiomyocyte cell death was assessed on different genetically modified models. In particular, an inducible tissue-specific *Mcu* deletion in the adult heart does not compromise the myocardial energy production and contractile function due to a compensatory increase in the fatty acid oxidation both under basal conditions and during the acute adrenergic challenge [117]. Moreover, such a deletion protects cardiomyocytes from mitochondrial Ca^2+^ overload and acute ischemia-reperfusion injury [95,96]. In line with these findings, the siRNA-mediated MCU silencing in vitro efficiently reduced mitochondrial Ca^2+^ overload, mPTP opening and cardiomyocyte death activation after hypoxia-reoxygenation [118]. On the other hand, myocardium-specific expression of a dominant negative form of MCU protein, induced before the birth, did not protect from the myocardial tissue death despite the preserved mitochondrial membrane potential and reduced ROS generation during ischemia/reperfusion [119]. These controversial observations led to a hypothesis that MCU deletions prior to birth result in compensatory changes in mitochondrial cell death pathways, and the protective effect of MCU depletion is dependent on the time of induction but not tissue specificity [120]. Indeed, a higher mPTP sensitivity to Ca^2+^ associated with the increased phosphorylation of cyclophilin D at Ser42 were observed in the hearts of conventional (whole-body) MCU KO mice, while the receptor interacting protein (RIP3) kinase-activated cell death pathway had no significant contribution to ischemia/reperfusion injury [120]. 

Conductance of mtCU channel is tightly regulated by auxiliary subunits; therefore, the resistance of cardiomyocytes to ischemia may be affected by alterations in either subunits expression or their association with MCU to form the whole mtCU complex. Indeed, the MICU1 protein content in mitochondria was significantly reduced following ischemia/reperfusion, despite the unchanged total MICU1 content, due to the inhibited expression of translocase of outer membrane 70 (Tom70) leading to the impaired MICU1 import into mitochondria [121]. Furthermore, targeting of myocardial MICU1 with siRNA significantly exacerbated the consequences of ischemic episode by increasing the infarction area, depressing cardiac function and increasing the myocardial apoptosis; the effects resulted from mitochondrial Ca^2+^ overload with consequent ATP depletion and morphological defects [121]. 

### 4.5. MtCU in Heart Failure

Heart failure is reported to be the prevalent reason for hospital admissions in some developed countries, and the imbalanced Ca^2+^-dependent regulation of oxidative metabolism is widely accepted as a key factor in its pathogenesis [122,123]. In particular, sustainably elevated cytoplasmic Ca^2+^ concentrations resulting from decreased SERCA expression or activity, along with Ca^2+^ leakage via RyR2, may lead to mitochondrial Ca^2+^ overload [1]. This, in turn, results in the elevated ROS levels and reduced ATP production, and eventually leads to maladaptive myocardium remodeling and contractile impairment [13]. Initial attempts to assess the physiological role of mtCU by using a conventional (whole-body) KO model surprisingly revealed that the absence of MCU expression has no influence on the cardiac morphology and function under basal conditions and is certainly insufficient to prevent the pressure overload-induced heart failure, despite the markedly impaired mitochondrial calcium handling [58]. At the same time, the isolated hearts of mice with myocardium-delimited transgenic expression of a dominant negative form of MCU protein showed the increased oxygen consumption indicative of lower efficiency, despite the unaltered myocardium morphology and mitochondrial structure [119]. The lack of mtCU-mediated mitochondrial Ca^2+^ entry in cardiomyocytes could be compensated by changes in cytoplasmic Ca^2+^ homeostasis manifested as unchanged oxygen consumption by the isolated cardiac mitochondria [119]. Interestingly, the opposite situation is observed for the inducible cardiac-specific MCU deficiency: this model shows an increased cardiac output associated with the enhanced fatty acid oxidation, and the unchanged levels of glucose oxidation [117].

Heart failure progression is accompanied by an increase in the myocardial MCU protein content reaching the highest point during the compensatory hypertrophic phase. The MCU upregulation is accompanied by autophagy blockade, as indicated by an increase in the p62 (SQSTM1) protein content resulting from its reduced degradation [103]. Interestingly, pharmacological inhibition of mtCU restores the levels of autophagy and mitophagy, partially prevents both the cardiomyocytes hypertrophy and myocardial fibrosis, and alleviates the concomitant ventricular asynchrony [103]. On the other hand, correction of cardiac dysfunction by cardiac resynchronization therapy provides mitochondrial fission and autophagosome/mitophagosome formation underlying the alleviated cell enlargement and myocardial fibrosis [124]. This therapeutic approach leads to a significant reduction in MCU and p62 content accompanied by DRP1 upregulation [124], which brings up an important question of mtCU participation in the mitochondria quality control. Cho et al. have recently reported that mtCU mediates the DRP1-dependent Zn^2+^ influx into the mitochondrial intermembrane space during fission [125]. Although the compromised mitochondria quality control in heart failure has been documented [123], further studies are needed to clarify whether this mechanism is crucial for the heart failure progression.

In contrast to *Mcu−/−* mice, which show a mild phenotype, the *Micu2−/−* mice have increased left atrium diameter resulting from the retarded cardiomyocyte relaxation and consequent diastolic dysfunction [51]. These dysfunctional changes develop despite the compensatory enhanced SERCA expression and normal myocardial structure [51], emphasizing the important role of mtCU regulatory subunits and upstream regulatory molecules in the mitochondrial Ca^2+^ handling under pathological conditions. For instance, miR-1 is being extensively studied as a potential therapeutic target, given its contribution to cardiac hypertrophy [72] and remodeling [126] and its association with heart failure [127], whereas mtCU has been identified as a downstream target of miR-1 in the hypertrophic changes realization [72]. Nevertheless, prognostic value of miR-1/mtCU axis in the heart failure progression has not been assessed thoroughly. As far as the CaMKII/mtCU axis role in heart failure is concerned, although CaMKII has been shown to cause heart failure through the mtCU activation and mPTP opening leading to cardiomyocyte death [30], contradictory data about the direct regulation of mtCU by CaMKII [30,31,77] do not allow us to make unambiguous conclusions at this point until further investigation.

The imbalance in mitochondrial Ca^2+^ may also be associated with the disturbed Ca^2+^ efflux from mitochondria. The mitochondrial Na^+^/Ca^2+^ exchanger is essential for Ca^2+^ homeostasis, as its inducible deletion in mice leads to sudden death caused by myocardial dysfunction and heart failure [128]. The correct balance of non-mtCU molecules is therefore essential for the prevention of heart failure.

## 5. The Role of MtCU in Skeletal Muscle

### 5.1. General Role of MtCU in Skeletal Muscle

The major role of skeletal muscle is the conversion of chemical energy into mechanical movements necessary for the realization of many vital functions [129]; an increase of Ca^2+^ concentration in the sarcoplasm is essential for skeletal muscle contraction [130]. 

Since mitochondrial Ca^2+^ handling is crucial for the adaptation of energy production to the immediate energy demands of the muscle, and also provides a link between excitation and transcription, the mtCU function in skeletal muscle is widely investigated [130]. Large body of valuable data on the role of mtCU in skeletal muscle was obtained on models with mtCU deficiency (as in the case with myocardial tissue). The role of MCU as a trophic factor was initially revealed in adult mice with muscle-targeted MCU overexpression or silencing as a cause of muscle hypertrophy or atrophy, respectively [131]; these findings were consistent with the smaller size of muscle fibers in muscle-targeted MCU KO mice [131,132] and the decreased body weight of conventional (whole-body) MCU KO mice [133]. However, more recent study did not show direct influence of the muscle-specific *Mcu* depletion on body weight, muscle weightm and fiber size regardless of the age of gene loss induction [134]. Some discrepancies between the models are also observed for the basal mitochondrial Ca^2+^ level. Whereas the basal mitochondrial Ca^2+^ concentrations were substantially reduced in the whole-body KO and adult muscle-specific *Mcu* depletion models [131,133], in a skeletal muscle-specific loss-of-function model they were unaffected [134]. Nevertheless, the relevance of all models was confirmed by observations of the blunted mitochondrial Ca^2+^ uptake in skeletal muscle in response to different stimuli [131,132,133,134]. A metabolic shift toward fatty acid oxidation in skeletal muscle of MCU-deficient mice was reported [132,134]. Consistently, impaired glucose oxidation in these animals was observed due to a decreased activity of pyruvate dehydrogenase regulating the flow of primary glucose metabolites to mitochondrial oxidative phosphorylation [131,133]. Interestingly, the in vivo effects of *Mcu* depletion may also depend on the experimental approach. For instance, impaired performance of the animals in the intense muscle exercise tests was demonstrated for the whole-body [133] and muscle-specific *Mcu* deletion [132]. However, other authors observed similar impairments only when using the exercise protocol without a warm-up period [134]; after a long adaptation to running, the performance of KO mice was similar to the control group [134].

*Mcu* deletion in skeletal muscle tissue not only affects muscle metabolism, but also triggers systemic metabolic adaptations [132]. Indeed, gluconeogenesis enzymes were upregulated in the liver of mice with skeletal muscle-specific *Mcu* deletions. At the same time, reduced blood levels of glucose were accompanied by increased glucose uptake in muscles. Taken together, these observations indicate systemic metabolic adaptations allowing the liver to counteract excessive muscle glucose uptake and maintain euglycemia [132]. 

### 5.2. Specific Features of MtCU in Skeletal Muscle

Skeletal muscle tissue is one of the top energy consumers in the body and therefore has special needs of rapid and tightly controlled energy production. Since mitochondrial Ca^2+^ directs the conversion of nutrient energy into ATP by activating the Ca^2+^-dependent dehydrogenases of the tricarboxylic acid cycle, regulation of mitochondrial Ca^2+^ uptake is a crucial point for the proper muscular contractions [130]. An alternative splice variant of MICU1, called MICU1.1, forms a dimer with MICU2 (as MICU1 does) activating mtCU and promoting Ca^2+^ uptake at much lower cytosolic Ca^2+^ concentrations than conventional MICU1-MICU2 does. At low Ca^2+^ concentrations, MICU1.1 is also capable of gatekeeping. The effect is explained by the increased Ca^2+^ affinity of MICU1.1, which is an order of magnitude higher than that of MICU1, as estimated by isothermal titration calorimetry [47]. The exact mechanism of boosting of the EF-hand Ca^2+^ binding affinity by MICU1.1 extra exon remains obscure. Therefore, in this case alternative splicing works as a means of adaptation of mitochondrial Ca^2+^ uptake to energetic demands of a particular tissue.

### 5.3. MtCU in Skeletal Muscle Dysfunction, Injury and Ageing

MtCU dysfunction associated with MICU1 intronic insertions causing frameshifts and leading to significant downregulation of MICU1 mRNA levels has been identified. Clinical phenotype of these patients comprising proximal muscle weakness, learning difficulties and progressive extrapyramidal movement disorder [135]. In accordance with the mtCU gatekeeper role of MICU1 [41], cell cultures derived from these patients showed the enhanced Ca^2+^ uptake at low cytosolic Ca^2+^ concentrations and the damping of cytosolic Ca^2+^ signals due to increased mitochondrial Ca^2+^ buffering [135]. Moreover, increased rates of mitochondrial fission (mediated by the elevated activity of DRP1 [136] in the MICU1-deficient cells with chronically activated mtCU [135]) result in highly fragmented mitochondrial networks. This finding additionally illustrates the regulatory function of the uniporter in the mitochondrial quality control system. Further analysis of the identified mutant phenotypes revealed a compensatory increase in Ca^2+^ efflux via NLCX, counteracting the increased mitochondrial Ca^2+^ uptake [136]. As a result, the sodium-proton exchanger (NCX) is also activated, which attenuates the proton-motive force required for ATP synthesis. Eventually, these mechanisms cause muscle weakness concomitant with the mutations [136]. In line with this data, the loss of MICU1 protein expression due to deletion of exon 1 of MICU1 causes fatigue, lethargy, and weakness [137]. Mitochondrial Ca^2+^ uptake is also substantially impaired in the cells derived from these patients [137]. Surprisingly, the loss of MICU1 does not affect oxygen consumption or membrane potential [135,137]; this finding is consistent with the data for a MICU1 in vivo silencing murine model [53]. Another recent report focuses on novel nonsense MICU1 mutations [138], previously documented as minor alleles [139]. The authors identified 13 patients from Middle East who manifested characteristic symptoms of MICU1 deficiency, including muscle weakness and easy fatigability [138]. Overall, different MICU1 mutations lead to similar effects and severe phenotypes resembling mitochondrial diseases, which should be accounted for in diagnostics.

The beneficial role of mtCU-dependent mitochondrial Ca^2+^ uptake in skeletal muscles following muscle injury has also been demonstrated [140]. Specifically, Ca^2+^ activates mitochondrial ROS production which, in turn, facilitates the myocyte membrane repair by local activation of RhoA and triggering F-actin accumulation at the site of injury. Both mtCU inhibition and ROS quenching compromise the plasma membrane repair. Interestingly, this mechanism is common for myoblasts, mature skeletal myofibers, and nonmuscle cells [140]. These findings emphasize the role of mtCU in skeletal muscle repair and the positive role of ROS as valuable signaling molecules. 

Since mtCU has been characterized as a trophic factor in skeletal muscle, therapeutic approaches leading to increased mtCU expression or activity should be considered for the treatment of various atrophic muscle diseases, for example, age-related sarcopenia. Leg press exercise training and neuromuscular electrical stimulation of the anterior thigh quadriceps muscles beneficially affected the structure and function of the muscle tissue in 70-year-old sedentary volunteers [141]. In detail, the improvement in muscle strength and the recovery of the myofiber structure was accompanied by an increase in MCU protein expression without changes in the MCU mRNA levels (suggesting post-transcriptional regulation). Additionally, the expression of hypertrophy-associated genes (e.g., IGF1) was induced by the training, concomitantly with the inhibitory effect on atrophy-associated genes (e.g., MuRF1). It should be noted that neuromuscular electrical stimulation has a more profound effect than conventional physical exercise training, and may be considered as an alternative to the traditional rehabilitation therapy [141]. On the other hand, excessive expression of MCU in mice with impaired mitochondrial quality control contributes to mitochondrial Ca^2+^ overload, myocyte cell death, and skeletal muscle atrophy [74]. This effect is mediated by DRP1-miR1-MCU axis: *Drp1* inhibition downregulates miR1, leading to increased MCU protein levels. Simultaneously, MCU inhibition normalizes Ca^2+^ handling and ameliorates myofiber survival [74]. Altogether, these findings indicate the importance of proper Ca^2+^ balance, achieved by proper regulation of mtCU activity, for normal skeletal muscle functioning and prevention of muscular atrophy.

MtCU-associated function disturbances may be associated not only with structural and functional impairments of the complex itself, but also with its abnormal regulation. In particular, null mutations in the m-AAA protease *SPG7* in *Drosophila* lead to multiple degenerative changes, including skeletal muscle degeneration accompanied by accumulation of morphologically and functionally abnormal mitochondria [92]. Although this model recapitulates some features of the hereditary spastic paraplegia and could be relevant for understanding its molecular pathology, applicability of this model for development of new therapies for paraplegia requires proper validation.

## 6. MtCU in Smooth Muscle Pathologies

The role of mtCU in smooth muscle cells has been mainly investigated in terms of vascular smooth muscle physiology and dysfunction, since VSMCs are crucial players in the pathophysiology of hypertension, which contributes to the development of heart failure, myocardial infarction, stroke and other severe disorders [142]. For instance, studies on the influence of mtCU blockers on cerebral blood flow preceded the discovery of mtCU molecular components [143]. Nevertheless, the number of studies on the role of mtCU in smooth muscle is limited.

VSMC plasticity (the capability of the contractile-to-synthetic shift in phenotype) is pivotal for the vascular wall repair during the phase of cell migration and proliferation following injury. By contrast, if the vascular function is impaired chronically, as occurs in atherosclerosis and neointima formation, VSMC plasticity contributes to the pathology progression [144]. Mitochondrial Ca^2+^ uptake via mtCU is essential for mitochondrial mobility and the isolated aortic VSMCs migration during neointima formation, with CaMKII as an upstream regulator of this process [79]. The CaMKII or mtCU inhibition predictably abrogated VSMCs migration and mitochondrial translocation to the leading edge [79] suggesting this pathway as a novel therapeutic option to mitigate the neointimal hyperplasia. 

The excessive artery smooth muscle cells proliferation, migration, and apoptosis resistance are also typical of pulmonary arterial hypertension [70]. In contrast to aortic VSMCs, the pulmonary artery smooth muscle cells (PASMCs) from the patients with pulmonary arterial hypertension showed decreased content of mitochondrial Ca^2+^ in combination with increased content of cytosolic Ca^2+^ which contribute to PASMC proliferation and migration. PASMC Ca^2+^ disturbances are attributed to MCU downregulation and coordinated reciprocal upregulation of MICU1 resulting in the impaired mtCU function. Along the same line, inhibition of mtCU in normal PASMCs reproduced the pulmonary artery hypertension phenotype [69]. Disturbed mitochondrial Ca^2+^ handling was also identified for maternally-inherited hypertension associated with a mutation in mitochondrial DNA [145]. In particular, Chen et al. revealed the lowest mitochondrial Ca^2+^ in combination with the highest cytoplasmic Ca^2+^ in lymphoblastoid cell lines derived from hypertensive patients carrying the mitochondrial DNA mutation in comparison with both hypertensive patients without the mutation and normotensive carriers of the mutation. The decreased MCU expression was characteristic of the cells from hypertensive patients both with mutation and without it [145]. Unfortunately, the paper provides neither mechanistic insights nor matching experiments with VSMCs.

The hypertension, induced by chronic infusion of angiotensin II in the *Micu2*-depleted mice, led to a progressive increase in the aortic diameter and eventually to aneurysms in some animals, although the initial aortic diameter in *Micu2−/−* mice was only slightly larger than in normal animals. Interestingly, this effect was specific for angiotensin II and was not reproduced by the treatment of *Micu2−/−* animals with norepinephrine. Transcriptional profiling of aortic VSMCs revealed dysregulation including proinflammatory signature, increased extracellular matrix remodeling, and cell proliferation [51]. Therefore, genetic defects in the mtCU subunit-encoding genes can exacerbate the adverse effects of cardiovascular pathologies. Although the issue is beyond the scope of this review, endothelial dysfunction contributing to the disease progression should be also taken into consideration when discussing hypertension. 

The data concerning mtCU function in the smooth muscle from non-vascular locations are extremely scarce and indirect, although the mitochondrial Ca^2+^ mishandling in smooth muscle tissue is typical for a number of diseases. For instance, hyper-responsiveness of the airway smooth muscle in asthma results from the elevated cytoplasmic Ca^2+^ very probably associated with dysregulated mitochondrial Ca^2+^ buffering [146]. Inhibitory analysis indicated the mtCU-mediated mitochondrial Ca^2+^ buffering of cytosolic Ca^2+^ elevations in the histamine-stimulated airway smooth muscle cells [147], suggesting the involvement of mitochondrial Ca^2+^ buffering in shaping of cytoplasmic Ca^2+^ responses. Interestingly, the observed differential responses of perinuclear and peripheral mitochondrial populations indicate the tight control of local Ca^2+^ concentrations and adaptation to specific energy demands in different cellular compartments [147]. Expression of mtCU components in myometrium along the course of human pregnancy has been recently analyzed [148]. The opposite dynamics between mRNA and protein expression was revealed for MCU and MCUb subunits: An abrupt transcriptional decline in the whole-term pregnancy was accompanied by increase in the corresponding protein levels. This pattern may be associated with the myometrial hypertrophy during gestation or with preparation to coordinated contractions at childbirth [148]. Overall, the role of mtCU in smooth muscle physiology and pathology remains largely understudied and further findings in this field are expected. 

## 7. Future Directions and Conclusions

MtCU is the major route for the Ca^2+^ influx to mitochondria, and its contribution to pathophysiology has been demonstrated for a number of cardiovascular and skeletal muscle diseases. Therefore, modulation of mtCU expression and activity represents a promising molecular target. Ruthenium red and its derivatives are usually used for inhibition of mtCU in the experiments. However, ruthenium red, along with the inhibition of mtCU, has nonspecific activity against some other ion channels [149], which prevents its use as a therapeutic agent. Use of the more selective ruthenium red derivative, Ru360, is also limited as it is plasma membrane-impermeable, and its synthesis is challenging [150]. Two cell-permeable and highly selective small-molecule mtCU inhibitors were recently developed: DS16570511 [151] and ruthenium complex Ru265 [150]. DS16570511 effectively blocks Ca^2+^ overload and increases cardiac contractility without affecting heart rate [151]. Ru265 is minimally toxic and prevents hypoxia/reoxygenation injury in cell model [150]. Anti-cancer drug mitoxantrone effectively inhibits mtCU activity, but its therapeutic use is limited by its cytotoxicity [152]. MtCU activators should also be considered as therapeutic agents for mtCU-associated pathologies. For instance, kaempferol, widely studied for its anti-cancer [153] and cardioprotective [154] properties, could be a promising agent in the prevention of Ca^2+^-triggered arrhythmias [110]. Even though these compounds have a prominent effect on mtCU-associated pathologies and may be promising drugs, they require more detailed validation in cardiovascular and muscle disease animal models before being introduced into clinical practice.

The mitochondrial Ca^2+^ uptake via mtCU is a crucial process for the life of a cell. The controllable influx of Ca^2+^ ensures proper energy production and its adaptation to the immediate needs in the tissues with high energy demands, such as cardiac and skeletal muscle tissues. In addition to the energy production, mitochondrial Ca^2+^ regulates a variety of myocyte functions including trophism, cell division, and cell death. MtCU expression and activity are tightly regulated by the number of intracellular signaling pathways, with some peculiarities in different types of muscle tissues. Disruptions in mitochondrial Ca^2+^ handling significantly increase the risks of cell and tissue dysfunction and ultimately result in the development of different pathologies. Cardiovascular diseases, currently known as one of the leading causes of death, and some skeletal muscle pathologies are shown to be associated with mtCU dysfunction. Therefore, a better understanding of fundamental mechanisms of mtCU function and dysfunction in muscle tissues may be useful for the future development of new modulators of mtCU activity and perspective therapy approaches.

## Abbreviations

CaMKII	calmodulin-dependent protein kinase II
CCD	coiled-coil domain
CPVT	catecholaminergic polymorphic ventricular tachycardia
CREB	cyclic AMP response element binding protein
cryo-EM	cryo-electron microscopy
DRP1	dynamin-related protein 1
EMRE	essential MCU regulator
KO	knockout
MCU	mitochondrial calcium uniporter pore-forming subunit
MICU1	mitochondrial calcium uptake 1
MICU2	mitochondrial calcium uptake 2
miR	microRNA
mtCU	mitochondrial calcium uniporter complex
NCX	Na+/Ca^2+^ exchanger
NMR	nuclear magnetic resonance
NTD	N-terminal domain
PASMC	pulmonary artery smooth muscle cells
PRMT1	protein arginine methyl transferase 1
RIPK1	receptor-interacting protein kinase 1
ROS	reactive oxygen species
RyR2	ryanodine receptor 2
SERCA	sarcoplasmic/endoplasmic reticulum Ca^2+^ ATPase
SR	sarcoplasmic reticulum
TMD	transmembrane domain
VSMCs	vascular smooth muscle cells
